# Near-complete genome sequence of nervous necrosis virus from big-belly seahorses (*Hippocampus abdominalis*) collected in China

**DOI:** 10.1128/mra.00162-25

**Published:** 2025-06-11

**Authors:** Xiaogang Yang, Meisheng Yi, Ming Li, Kuntong jia

**Affiliations:** 1 School of Marine Sciences, Sun Yat-Sen University 26469 , Guangzhou, Guangdong, China; 2 Southern Marine Science and Engineering Guangdong Laboratory (Zhuhai) 590852 , Zhuhai, Guangdong, China; 3 Guangdong Provincial Key Laboratory of Marine Resources and Coastal Engineering, Guangzhou, China; 4 Guang Xi Key Laboratory of Aquatic Genetic Breeding and Healthy Aquaculture, Guang Xi Institute of Fisheries 612477 , Nanning, Guangxi, China; Katholieke Universiteit Leuven, Leuven, Belgium

**Keywords:** *Hippocampus abdominalis*, nervous necrosis virus, RNA virus, sequencing

## Abstract

A nervous necrosis virus (NNV) strain was detected from big-belly seahorses (*Hippocampus abdominalis*) in China in 2024. The near-complete genome sequence (4,689 base pairs, 46.11% GC content) is closely related to other marine fish NNV isolates. The data aid in understanding NNV diversity and support diagnostic and vaccine development.

## ANNOUNCEMENT

Nervous necrosis virus (NNV), a member of the *Betanodavirus* genus (*Nodaviridae*) (1), is an unenveloped icosahedral RNA virus composed of two positive-sense RNA segments, RNA1 and RNA2, which encode RNA-dependent RNA polymerase (RdRp) and capsid protein (CP), respectively. NNV causes severe viral nervous necrosis in aquatic species globally, despite existing control measures (2–9). While two formalin-inactivated vaccines (*Alpha ject micro 1Noda* [Pharmaq] and Icthiovac *VNN* [Hipra]) target the RGNNV genotype in sea bass, continuous viral evolution threatens vaccine efficacy (7). Comprehensive genomic surveillance of NNV strains is thus critical to track viral dissemination and guide countermeasures.

**Fig 1:**
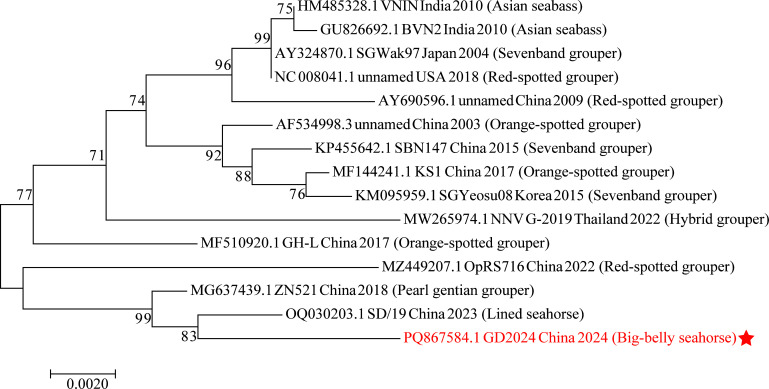
Neighbor-joining (NJ) phylogenetic analysis of 15 complete nucleotide sequences of capsid protein (CP) with 1,000 bootstrap replicates in MEGA v7.0 with default parameters. Globally representative reference sequences of NNV were randomly selected and downloaded from GenBank through the NCBI. The data set for each virus was aligned using Muscle in Mega v7.0 (16). Previously aligned sequences were trimmed to equal lengths. Each terminal node was assigned a GenBank accession number, followed by the strain name and geographic location. Hosts of viral strains are shown in parentheses. The strain GD2024 in this study is marked with a red color.

Here, we sequenced the genome of an NNV strain detected from farmed big-belly seahorses (*Hippocampus abdominalis*) in Guangzhou, China. Symptomatic seahorses (erratic swimming, darkened pigmentation, anorexia) were collected from a mariculture facility on September 15, 2024. Brain and eye tissues were homogenized for 30 s in 1.5 mL centrifuge tubes containing phosphate-buffered saline (1×, pH 7.4), centrifuged at 15,000 × *g* for 3 min at 4°C, and viral RNA was extracted using the E.Z.N.A. Viral RNA Kit (Omega). Successful amplification of the capsid protein gene (25 PCR cycles) was evidenced by bright bands, confirming the presence of NNV with high viral load prior to sequencing (10). For this purpose, RNA was reverse-transcribed using the HiScript 1st Strand cDNA Synthesis Kit (Vazyme) with random hexamers after DNase treatment. PCR amplification utilized NNV-specific primers (F: 5′-GTCGGCTGATACTCCTGTGTG-3′; R: 5′-CTCCAGTTCCAAGGCTGTAGT-3′) under optimized conditions: 98°C, 1 min initial denaturation; 25 cycles of 98°C, 30 s, 55°C, 10 s, 72°C, 20 s; final extension at 72°C, 2 min (Q5 High-Fidelity PCR Kit). cDNA was also used to prepare libraries with the TruSeq Nano DNA Library Prep Kit (Illumina) and sequenced on the NovaSeq 6000 platform (2 × 150 bp paired-end reads). Read quality trimming was performed using fastp v0.20.0 (11) with an additional trimming filter for adapters and low-quality reads, including those reads scored <Q20. Sequencing reads were filtered using BBmap v38.51 (12) to remove host (*Hippocampus abdominalis* genome: GCA_018466805.1), bacterial (NCBI RefSeq complete genomes), and contaminant sequences (rRNAs: 16S, 18S, 23S, 28S, 5S; mitochondrial DNA). *De novo* assembly was performed using SPAdes v3.14.1 (13), and the resulting contigs were aligned to reference genomes *Dragon grouper nervous necrosis virus* (GenBank accession: AY721616.1) and *Pearl gentian grouper nervous necrosis virus* (GenBank accession: MG637439.1) using BLAST+ v2.10.0 (14). The final assembly of all data resulted in 325 contigs over 1,000 bp in length. Genome annotation was carried out using Prokka v1.14.5 (15). For all tools, default parameters were used except where otherwise noted. The near-complete genome contains two expected viral proteins: capsid protein (CP) (1489 bp) and RNA-dependent RNA polymerase (RdRp) (3,200 bp), which shared 99.1% nucleotide identity with NNV SD/19 and 99.2% with IZSM 103888 Italy 2024 (GenBank accession: PQ871107.1), respectively. Phylogenetically, the CP gene of our strain clustered most closely with NNV SD/19 China 2023 (*Hippocampus erectus*, GenBank accessions: OQ030203.1) (Fig. 1).

In conclusion, this near-complete genome sequence of NNV detected from *Hippocampus abdominalis* provides valuable insights into the virus’s genetic structure and evolution.

## Data Availability

The genome sequence has been deposited in GenBank under accession numbers PQ867583.1 and PQ867584.1. All raw sequencing reads are available in the NCBI Sequence Read Archive (SRA) under the accession number SRR32438484.
